# Real-time and single shot CMR increases throughput and improves reliability

**DOI:** 10.1186/1532-429X-14-S1-P43

**Published:** 2012-02-01

**Authors:** Abdul Wattar, Subha V Raman, Orlando P Simonetti

**Affiliations:** 1 Ohio State University, Columbus, OH, USA

## Summary

The purpose of this study is to compare exam time and quality of images obtained using standard segmented k-space acquisitions versus single-shot and real time techniques that require no breath-holds.

## Background

The typical CMR exam utilizes segmented k-space acquisitions that require repeated breath-holds, regular cardiac rhythm, and total exam times on the order of forty-five minutes. Widespread utilization of CMR has been hampered by these prolonged exam times and limited reliability in patients with irregular rhythm and/or inability to breath-hold.

## Methods

Cardiac MRI studies performed at our lab over a twenty-five day period were retrospectively reviewed and 16 consecutive studies identified that did not involve stress imaging or MR angiography; the majority included cine, late gadolinium enhancement (LGE), and velocity mapping. Indications for CMR were: cardiomyopathy 11, cardiac mass 2, valvular 1, viability 1, pericardial 1. Studies were separated into two groups: eight were performed using standard segmented k-space acquisitions and eight in which cardiac arrhythmia or inability to breath-hold led to the use of real-time and single-shot methods exclusively. All images were qualitatively assessed for image quality, ability to estimate function.

## Results

Total exam time for patients scanned using the standard breath-hold protocols was (mean+/- SD) 37.1+/- 13.5 minutes compared with 26.6 +/- 7.6 minutes for patients scanned using real-time cine and velocity-mapping and single-shot LGE methods. In patients where segmented acquisitions were attempted but abandoned due to arrhythmia or inability to breath-hold, real-time imaging significantly reduced artifacts and cardiac endocardial borders were better identified compared to segmented scans under these circumstances. LV wall motion assessment was feasible in seven out of the eight patients who had to undergo the real time cine imaging (87.5%) vs. all eight patients (100%) who underwent standard segmented cine imaging.

## Conclusions

Real-time and single-shot imaging not only reduces scan time, but also improves reliability in patients with arrhythmias or inability to breath-hold. The use of real time cine in these patients did not compromise the ability to analyze function and morphology and provided proper diagnostic answers. A prospective cohort study is underway to evaluate the reliability and cost-effectiveness of such an approach.

## Funding

None.

**Figure 1:**
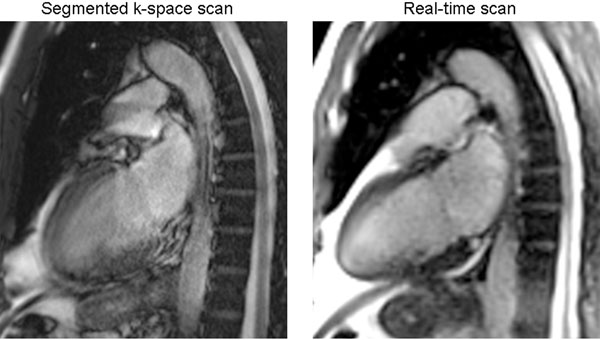
Single frame from standard segmented k-space cine (Left) and real-time cine (Right) scans in patient with severe arrhythmia. Significant blurring of myocardial wall observed in segmented acquisition while real-time scan is impervious to arrhythmia.

